# E1231/NMN protects against experimental metabolic syndrome: the central role of SIRT1 in modulating AKT/Nrf2/NFκB signaling

**DOI:** 10.3389/fphar.2025.1558709

**Published:** 2025-03-17

**Authors:** Elsayed A. Elmorsy, Hala A. Elashry, Abdullah S. Alkhamiss, Hamad Alsaykhan, Rabab S. Hamad, Mustafa Ahmed Abdel-Reheim, Mansour Alsoghair, Mariam S. Alharbi, Attia M. Gabr, Abousree T. Ellethy, Mostafa M. Khodeir, Ageeb M. Hassan, Hossam A. Elsisi, Alshaimaa A. Farrag, Norah Suliman Alsoqih, Ahmed Sameh, Sameh Saber

**Affiliations:** ^1^ Department of Pharmacology and Toxicology, College of Pharmacy, Qassim University, Buraidah, Saudi Arabia; ^2^ Department of Clinical Pharmacology, Faculty of Medicine, Mansoura University, Mansoura, Egypt; ^3^ Department of Pathology, College of Medicine, Qassim University, Buraidah, Saudi Arabia; ^4^ Department of Anatomy and Histology, College of Medicine, Qassim University, Buraidah, Saudi Arabia; ^5^ Biological Sciences Department, College of Science, King Faisal University, Al Ahsa, Saudi Arabia; ^6^ Department of Pharmacology, College of Pharmacy, Shaqra University, Shaqra, Saudi Arabia; ^7^ Department of Family and Community Medicine, College of Medicine, Qassim University, Buraidah, Saudi Arabia; ^8^ Department of Medicine, College of Medicine, Qassim University, Buraidah, Saudi Arabia; ^9^ Department of Basic Oral Sciences and Dental Education, Biochemistry Division, College of Dentistry, Qassim University, Buraidah, Saudi Arabia; ^10^ Department of Pathology, Faculty of Medicine, Cairo University, Cairo, Egypt; ^11^ Department of Clinical Pharmacology, Faculty of Medicine, Zagazig University, Zagazig, Egypt; ^12^ Department of Anatomy, College of Medicine, University of Bisha, Bisha, Saudi Arabia; ^13^ Department of Pediatrics, College of Medicine, Qassim University, Buraidah, Saudi Arabia; ^14^ Faculty of Computing and Information Sciences, Egypt University of Informatics, Cairo, Egypt; ^15^ Department of Pharmacology, Faculty of Pharmacy, Delta University for Science and Technology, Gamasa, Egypt

**Keywords:** E1231, EX527, SIRT1, metabolic syndrome, AKT/Nrf2/NFκB, insulin resistance

## Abstract

Metabolic syndrome (MetS) is a cluster of several disorders where many challenges hinder effective treatment. The downregulation of SIRT1 or inhibition of its activity is implicated in its pathophysiology. We hypothesized that the combined SIRT1 direct activator E1231 and the SIRT1 stabilizer nicotinamide mononucleotide (NMN) could offer a novel approach to mitigate the pathophysiological features of MetS. Our results revealed that E1231 alone or combined with NMN increased SIRT1 level and activity. This SIRT1 activation was accompanied by upregulation in the IRS-1 and activation of AKT. In parallel, the Nrf2 level and activity were increased while the NFκB activity and subsequent inflammatory cytokines were decreased. Additionally, SIRT1 activation was associated with improved insulin resistance, blood pressure, lipid profile, fasting blood glucose, glucose tolerance, and kidney and liver functions. Moreover, improved liver histology, decreased hepatic fibrosis markers, and increased survival rates were observed. These protective functions were counteracted when EX527, a SIRT1 inhibitor, was dually administered with E1231. Furthermore, correlation analysis revealed that SIRT1 was negatively correlated with NFκB, insulin resistance, and oxidative stress, while positive correlations were observed between SIRT1, p-AKT, and Nrf2 activity. Random Forest regression algorithm and partial dependence plots highlighted the significant roles of SIRT1, IRS-1, p-AKT, and NFκB in predicting MetS severity. These analyses underscore the strong interconnections between these signals. This reinforces the central role of SIRT1 in coordinating a multifaceted protective response against MetS. To conclude, SIRT1 alleviates MetS by modulating AKT/Nrf2/NFκB signaling and their interactions. Further research is necessary to validate these findings.

## 1 Introduction

Metabolic syndrome is a cluster of various disorders that increase the risk of type 2 diabetes and other associated complications (Mohamed et al., 2023). Globally, the prevalence and incidence of the metabolic syndrome are radically rising (Chooi et al., 2019). Factors that determine this syndrome comprise insulin resistance, central obesity, high blood pressure, and dyslipidemia (Jha et al., 2023). Several challenges hinder effective therapy and outcomes. These include polypharmacy, variable response to treatment, side effects, and tolerability. Moreover, due to insufficient targeting, current therapeutic modalities may not address the underlying pathophysiology (Aguilar-Salinas and Viveros-Ruiz, 2019).

Sirtuin 1 (SIRT1) is a protein of the nicotinamide adenine dinucleotide (NAD^+^)-dependent deacetylases (Tanno et al., 2007). Sirtuins are fundamental regulators of various cellular processes, such as stress and energy metabolism (Li, 2013a). SIRT1 exerts its function by deacetylating transcription factors and co-factors (Li, 2013b). Elevation of SIRT1 has been known to have beneficial effects in mice fed a high-fat diet (HFD) (Guéant et al., 2014). In contrast, the downregulation of SIRT1 or the inhibition of its activity has been implicated in the pathophysiology of metabolic syndrome determinants, including insulin resistance, type 2 diabetes, and liver lipid accumulation (Wang et al., 2012). It is noteworthy that a reduction in SIRT1 has been linked to overnutrition or exposure to an HFD (Kosgei et al., 2020). Understanding this function provides insights into the potential benefits of sirtuin activators in metabolic syndrome. Activators of SIRT1 might have therapeutic potential in treating metabolic syndrome-related outcomes, although results have not been acknowledged to date.

E1231, C21H21N3O3, was designed as an orally active SIRT1 activator. It has demonstrated a capacity to regulate cholesterol and lipid metabolism in golden hamsters fed with an HFD and in *ApoE* ^−/−^ mice fed with an atherogenic diet (Feng et al., 2018). It was also demonstrated that E1231 might be effective in halting the development of non-alcoholic steatohepatitis (NAFLD) (Han et al., 2023). The possible benefit of NAD^+^ supplementation in stabilizing the activity of SIRT1 has been recognized by Cantó and Auwerx (2012). Notably, nicotinamide mononucleotide (NMN) is one of the main precursors of NAD^+^ that has been uncovered to improve health and lifespan with great tolerability and safety (Yi et al., 2023). In this regard, our study hypothesized that supplements aimed at increasing NAD^+^ availability may stabilize and boost SIRT1 activity. This might lead to improved metabolic syndrome outcomes. Therefore, it is proper to claim that NMN can be a candidate adjuvant to augment and enhance the E1231-induced SIRT1 activity.

Protein kinase B, which is also known as AKT, plays a crucial role in the insulin signaling cascade. The activation of AKT leads to an increase in glycogenesis and a reduction in gluconeogenesis and glycogenolysis in hepatic tissue (Savova et al., 2023). The nuclear factor erythroid 2-related factor 2 (Nrf2) is a well-known transcription factor modulator of the antioxidant response. It triggers the transcription of antioxidant and detoxifying genes that counteract oxidative stress (Reed et al., 2024). The nuclear transcription factor kappa B (NFκB) is a pro-inflammatory transcription factor. Metabolic stresses like elevated glucose and fatty acid levels chronically activate NFκB. This maintains a chronic inflammatory state by producing inflammatory cytokines such as TNF-α and IL-6 (Dali-Youcef et al., 2013). It should be noted that extensive crosstalk between the AKT, Nrf2, and NFκB pathways might exist (Elbaset et al., 2024). This signaling interaction could potentially contribute to the pathology of metabolic syndrome. Additionally, dysregulated crosstalk between AKT, Nrf2, and NFκB signaling engages a fundamental role in the development and progression of metabolic syndrome by augmenting oxidative stress and inflammation (Salminen et al., 2011; Liu et al., 2016; Kracht et al., 2020). Hence, balancing these pathways and controlling their interaction, in particular the improvement of Nrf2 activity and the suppression of NFκB, has emerged as a promising strategy for managing metabolic syndrome.

This study is the first to investigate the potential protective role of combined treatment with E1231 acting as a direct SIRT1 activator and NMN acting as an SIRT1 stabilizer on metabolic syndrome. The study, in a rat model induced by STZ and a high-fat diet, seeks to assess whether the combined therapy of a SIRT1 direct activator along with a SIRT1 stabilizer can offer an approach to alleviate the pathophysiological features of metabolic syndrome, including insulin resistance, oxidative stress, inflammation, dyslipidemia, and hepatic lipid accumulation. This study also aims to evaluate the interconnectedness between key metabolic and molecular parameters through correlation analysis. Moreover, the study identifies the most influential predictors of metabolic syndrome severity using Random Forest regression machine learning algorithm and Partial Dependence plot analyses. Furthermore, the study investigates the potential effects of SIRT1 activation on HFD-induced weight gain over weeks.

## 2 Methods

### 2.1 Animals

Delta University’s research ethics committee approved the study design conducted on male adult Sprague Dawley rats (200 ± 10 g), which were purchased from Delta University’s animal facility, Egypt; approval number 16/2022,8. Guidelines were followed during the acclimation and experimental phases. Rats were allowed free access to water and either a standard diet (SD) or a high-fat diet (HFD). Sample size was selected based on pilot study followed by power analysis using G*power 3.1.9.7, which considered the α level, sample size, and effect size.

### 2.2 Experimental diet

The composition of the experimental diet is described in Table 1, as previously reported (Lomba et al., 2010; Yeung, 2015; Keshk et al., 2020). El-Kahira Company for oils and soap, Egypt, supplied the SD or HFD as rodent chow.

**TABLE 1:** Experimental diet.

Nutrient	Standard diet	High-fat diet
Protein	20%	20%
Carbohydrates	67%	35%
- Starch	62%	10%
- Sucrose	5%	18%
- Maltodextrin	-	7%
Fat	13%	45%

### 2.3 Materials, reagents and kits

Streptozotocin (STZ) was purchased from Sigma-Aldrich (St. Louis, MO, United States). Sodium citrate buffer was purchased from El Gomhouria, Cairo, Egypt. E1231 was obtained from MCE, NJ, United States NMN and glutathione peroxidase assay kits were purchased from Sigma-Aldrich. EX527 was supplied by Tocris Bioscience, Bristol, United Kingdom.

The systolic blood pressure (SBP) was assessed in rats utilizing a tail-cuff sphygmomanometer. Fasting blood glucose (FBG) levels were checked using a GlucoDr glucometer. The oral glucose tolerance test (OGTT) was performed using a 2 g/kg glucose solution. The quantitative insulin sensitivity check index (QUICKI) was determined using the equation: QUICKI = 1/(log FBI (mU/L)) + log FBG (mg/dL)) (Katz et al., 2000). The homeostatic model assessment of insulin resistance (HOMA-IR) was driven utilizing the equation: HOMA-IR = fasting serum insulin (FSI) (mU/L) × FBG (mg/dL)/405, as reported (Matthews et al., 1985).

Reagent kits for serum creatinine, urea, microalbumin, liver enzyme activities (alanine aminotransferase (ALT), aspartate aminotransferase (AST), alkaline phosphatase (ALP), gamma-glutamyl transferase (γGT), and antioxidant markers (glutathione (GSH), superoxide dismutase (SOD), catalase (CAT)) were provided by Biodiagnostic, Giza, Egypt. Levels of serum total cholesterol (TC) and triglycerides (TG) were measured using commercially available kits from Biodiagnostic. Low-density lipoproteins (LDL) were calculated using the Friedewald equation: LDL (mg/dL) = TC (mg/dL)-HDL (mg/dL)-(TG (mg/dL)/5) (Friedewald et al., 1972). Very Low-Density Lipoprotein (VLDL) was estimated using the equation TG/5. The content of TC and TG in the liver was determined using kits obtained from Abcam (MA, United States).

ELISA kits were employed for assessing FSI (Cloud-Clone Corp., China), kidney injury molecule 1 (Kim-1) and heme oxygenase 1 (HO-1) (MyBioSource, CA, United States), TNF-α (Enzo Life Sciences, Germany), IL-6 (R&D Systems, United States), transforming growth factor-beta 1 (TGF-β) (eBioscience, Austria), tissue inhibitor of metalloproteinase (TIMP-1) and p-Ser473 AKT (RayBiotech, United States). Hydroxyproline content was determined using reagents from Sigma-Aldrich (Reddy and Enwemeka, 1996). NFκB activity, SIRT1 expression, and activity levels were measured using kits from Abcam and Cloud-Clone Corp (Houston, TX, United States), respectively. Nrf2 expression and activity were assessed using a MyBioSource (CA, United States) kit, with nuclear translocation detected through Abcam antibodies. All assays were performed following the manufacturer’s instructions.

### 2.4 Experimental design

The control group (n = 8) was fed SD for 12 weeks. The E + N group (n = 8) was fed SD for 12 weeks and received E1231 and NMN during the last 8 weeks, while the E + EX group (n = 8) was on an SD for 12 weeks and received E1231 + EX527 during the last 8 weeks.

Rats were fed HFD for 4 weeks to induce animal metabolic syndrome experimentally. Then, animals were administered streptozotocin (STZ) at a dose of 40 mg/kg, IP dissolved in sodium citrate buffer (pH 4.4). Rats having Fasting blood glucose (FBG) levels above 200 mg/dL after 72 h were categorized as diabetic. These diabetic rats were then kept on HFD for a further 8 weeks (Huang et al., 2020). These rats were divided into four groups: The MetS (n = 10) is a control diabetic group that maintained feeding on HFD for 12 weeks. The MetS/E (n = 8) is a diabetic group that maintained feeding on HFD for 12 weeks and treated with E1231. The MetS/E + N (n = 8) is a diabetic group that maintained feeding on HFD for 12 weeks and was administered both E1231 and NMN. MetS/E + EX (n = 8) is a diabetic group that maintained feeding on HFD for 12 weeks and was administered both E1231 and EX527.

**FIGURE 1:**
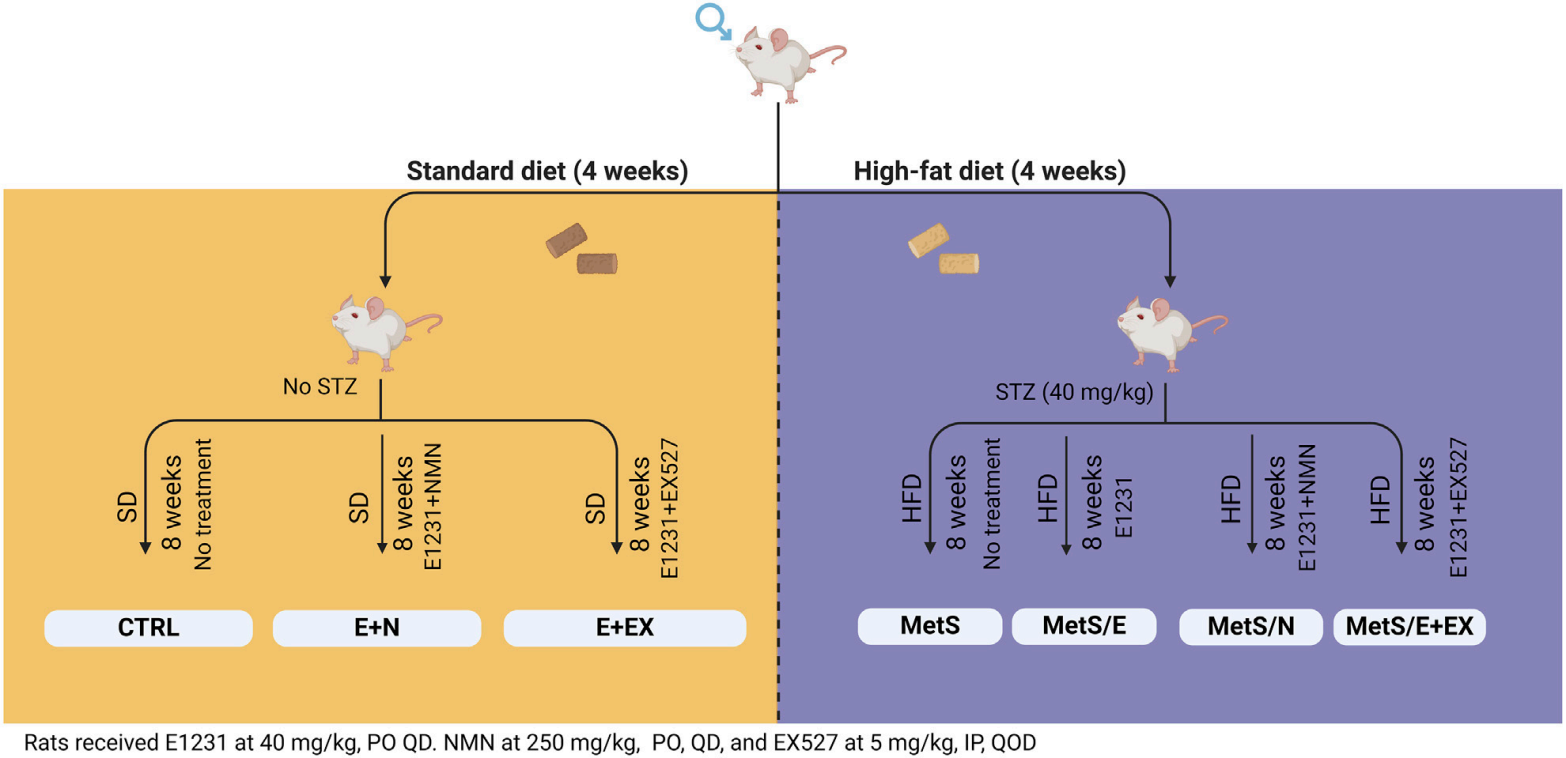
Schematic illustration of experimental protocol.

Rats received interventions during the final 8 weeks of the experiment. E1231 was prepared by dissolving in 0.5% CMC and was administered at 40 mg/kg/day, PO, as reported (Feng et al., 2018; Han et al., 2023). NMN was prepared by dissolving in 0.5% CMC and was administered at 250 mg/kg/day, PO, as reported (Huang et al., 2023). EX527 was prepared by dissolving in saline containing 1% DMSO and was administered every 2 days at a dose of 5 mg/kg, IP (Smith et al., 2014; Ma et al., 2020). NMN or EX527 was administered 1 hour before E1231. All groups received the same vehicles. The Experiment protocol is displayed in Figure 1. Two days before the end of the experiment, groups fasted overnight. Then, OGTT was performed. At the end of the experiment, groups fasted overnight, and anesthesia was induced with secobarbital (50 mg/kg, IP). Blood samples were collected via cardiac puncture.

### 2.5 Histological examination

Liver tissues were processed as per standard histological procedures and stained with hematoxylin and eosin (H&E) or Masson’s trichrome staining (Youssef et al., 2022). Following a blinded technique, a histopathologist examined 20 HPFs under Olympus CX23 light microscope (Tokyo, Japan). The scoring criteria used for histological evaluation are described in Table 2.

**TABLE 2:** Scoring Criteria used in histological examination.

Parameter	Score 0	Score 1	Score 2	Score 3	Score 4	Score 5	Score 6
Lobular inflammation	No inflammatory foci	Fewer than 2 foci per 200x field	2 to 4 foci per 200x field	More than 4 foci per 200x field	-	-	-
Steatosis	Less than 5% of hepatocytes affected	5%–32%	33%–66%	More than 66%	-	-	-
Collagen deposition	No fibrosis	Fibrous expansion of some portal areas with or without short septa	Fibrous expansion of most portal areas with or without short septa	Fibrous expansion of most portal areas with occasional portal-to-portal (P-P) bridging	Fibrous expansion with marked bridging (P-P and portal-to-central (P-C))	Marked bridging (P-P and/or P-C) with occasional nodules (incomplete cirrhosis)	Cirrhosis, probable or definite
Collagen area quantification	0% (No fibrosis)	< 5% (Normal to minimal fibrosis)	5-<15% (Mild fibrosis)	15-<25% (Moderate fibrosis)	25% or more (Severe fibrosis)	-	-

### 2.6 Statistical analysis

A dataset of parameters included p-AKT, IRS-1, SIRT1, Nrf2, NFκB, HOMA-IR, SBP, TG, and KIM-1 were used in the calculation of Pearson correlation coefficients and the generation of hierarchical clustered heatmap to show correlations. The heatmap was generated using Python’s Seaborn library. An interaction network map was created using Python’s Networkx library to visualize the complex relationships between biomarkers. The network displays nodes representing the biomarkers and edges representing significant correlations. The thickness indicates strength, and color intensity indicates the direction of the correlations. A scatter plot matrix was created using Python’s seaborn and matplotlib libraries to display the bivariate relationships between each pair of variables. This matrix allows for visually inspecting linear relationships, potential outliers, and trends. In this regard, the matrix’s diagonal elements display a variable’s distribution, and off-diagonal elements display the pairwise scatter plots. Statistical analysis was performed utilizing GraphPad Prism software (version 9.5.1, GraphPad Software, CA, United States), and the significance was set at p ≤ 0.05. The latter software was also used to generate Kaplan-Meier survival plots. Mantel-Cox log-rank tests were employed to compare survival between groups.

### 2.7 Assessment of body weight changes over 12 weeks

The percentage change in body weight over weeks was visualized using Python (version 3.11). The Matplotlib and Seaborn libraries were utilized to generate the plots. The percentage change in body weight for each group was calculated using the formula: percentage change = (final body weight – initial body weight)/initial body weight × 100. A heatmap was employed to demonstrate trends in percentage weight changes across weeks. The color intensities display the magnitude of such change. A line plot was generated to track longitudinal trends and visualize the percentage change in body weight over time. This can enable a clear comparison between different interventions and control groups. Additionally, a stacked area chart was utilized to display cumulative weight changes over the experimental time course. This highlights the contribution of each group to overall weight gain. Furthermore, the stacked area chart helped examine the effectiveness of the HFD in promoting weight gain as a measure of obesity.

### 2.8 Assessment of key metabolic and molecular determinants of metabolic syndrome

#### 2.8.1 Bubble chart visualization of key metabolic and molecular parameters affecting metabolic syndrome

A bubble chart was constructed as an approach to visualizing the effects of several analyzed metabolic and molecular parameters on HOMA-IR. This analysis was conducted using Python’s Pandas library for data manipulation and Matplotlib and Seaborn for visualization. The x-axis represents the parameter values, and the y-axis represents the HOMA-IR values. Bubble colors were distinctly utilized to differentiate different parameters. A larger bubble signifies higher HOMA-IR values and *vice versa*. This visualization uncovers the relative influence of the corresponding parameter on HOMA-IR as a determinant of metabolic syndrome.

#### 2.8.2 Random forest regression analysis of key metabolic and molecular predictors of metabolic syndrome

To verify factors involved in the pathophysiology of metabolic syndrome, a Random Forest machine learning algorithm was conducted to investigate the importance of various features (parameters) in predicting metabolic syndrome. Feature importance scores were extracted from the model to quantify the contribution of each variable parameter to the predictive power of the model. The feature importance plot was generated using Python’s Sklearn and Matplotlib libraries.

#### 2.8.3 Partial dependence plot analysis of key metabolic and molecular parameters influencing metabolic syndrome using a random forest model

Partial Dependence plots (PDPs) were constructed based on the Random Forest model. PDPs analyze the impact of different metabolic and molecular parameters on the predicted outcome. These plots also display the marginal effect of each parameter on the model’s predictions through the isolation of the effect of one variable while averaging out the influence of all other variables. The construction was conducted using Python’s sklearn package. In this regard, the PartialDependenceDisplay function was employed to visualize the relationship between each predictor and the predicted value. The y-axis represents the partial dependence, and the x-axis represents the range of each variable parameter. The Random Forest model was trained using the collected dataset. The resulting PDPs provide insights into how changes in individual parameters, such as increases in LDL or decreases in IRS-1, influence the model’s predictions.

## 3 Results

### 3.1 E1231 decreases hepatic inflammation, fibrosis, and steatosis in a rat model of metabolic syndrome

**FIGURE 2:**
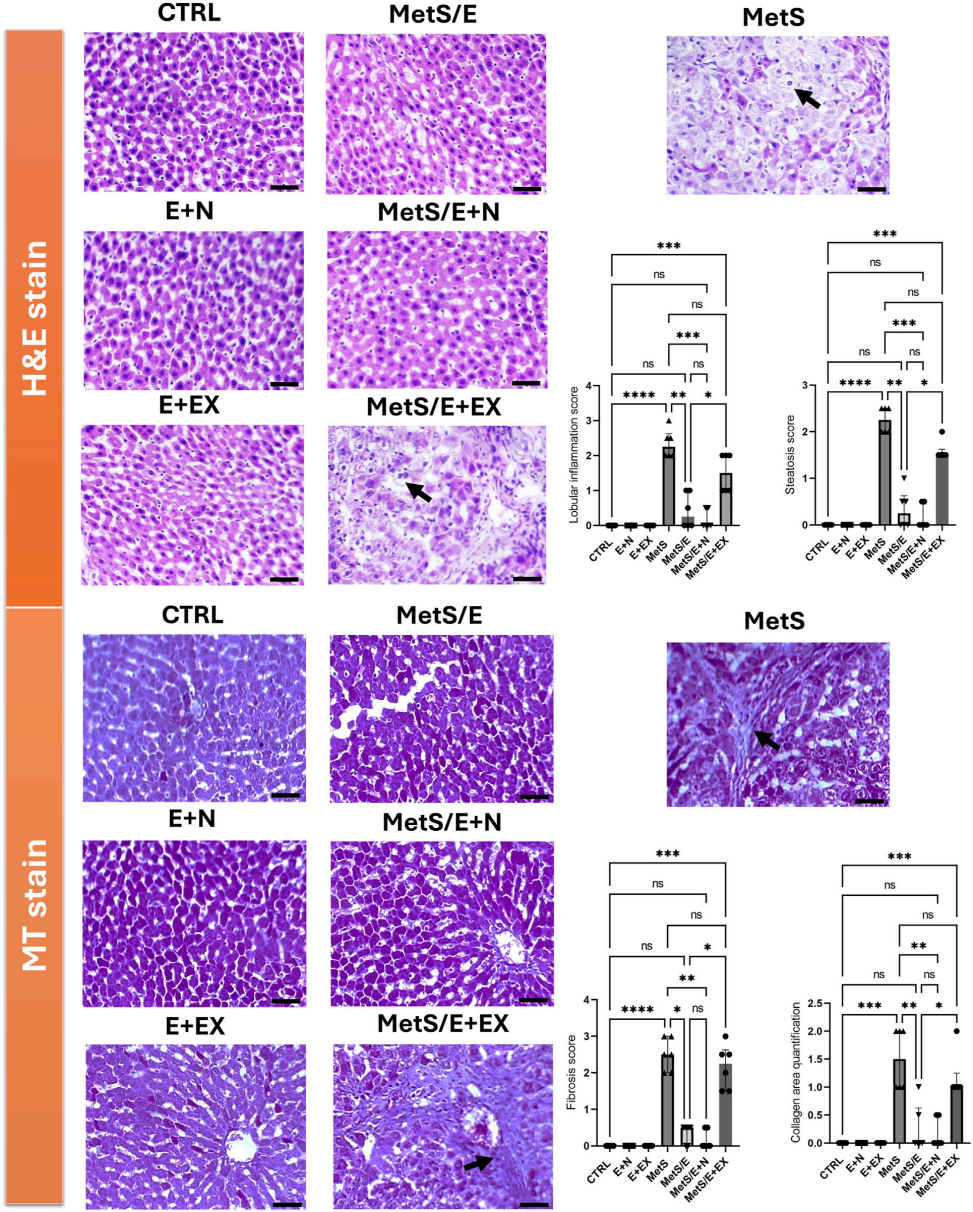
E1231 decreases hepatic inflammation, fibrosis, and steatosis in a rat model of metabolic syndrome. Histological analysis of liver sections stained with H&E in the upper panel and Masson’s trichrome (MT) in the lower panel shows that control groups maintain normal liver architecture with no significant steatosis, inflammation, or fibrosis. The MetS exhibits marked lobular inflammation, steatosis (arrow), and mild collagen deposition (arrow). The MetS/E displays attenuated pathological features. The MetS/E + N exhibits the most significant protection, as evidenced by the lowest scores in lobular inflammation, steatosis, fibrosis score, and collagen area quantification score. The MetS/E + EX does not show significant improvement compared to the MetS group with persistent steatosis (H&E panel, arrow) and fibrosis (MT panel, arrow). Statistical analysis was performed using the Kruskal–Wallis test followed by Dunn’s *post hoc* test, n = 6. *p < 0.05, **p < 0.01, ***p < 0.001, ****p < 0.0001.

As depicted in Figure 2, the upper panels display H&E-stained liver sections. The control groups show normal liver architecture with intact lobular structures. The MetS exhibits liver damage characterized by lobular inflammation and steatosis. Both parameters show high scores compared to the CTRL (p < 0.0001 for both parameters). However, MetS/E and MetS/E + N show varying degrees of improvement. In particular, the MetS/E + N group displays the most significant reduction in these scores (p < 0.001 for both parameters), displaying a protective effect of E1231 when combined with NMN. The MetS/E + EX demonstrates no significant improvement. It displays higher lobular inflammation (p = 0.42) and steatosis (p = 0.38) scores. The lower panels show liver sections stained with Masson’s trichrome. The control groups again exhibit normal liver architecture with no apparent collagen deposition. The MetS group shows increased collagen, indicative of mild fibrosis. In parallel, higher fibrosis scores and collagen area quantification compared to the CTRL were observed (p < 0.0001 and p < 0.001, respectively). The MetS/E and MetS/E + N significantly reduced fibrosis and collagen area quantification. Particularly, the MetS/E + N group reveals enhanced protection. The fibrosis and collagen area quantification scores are significantly lower in the MetS/E + N group compared to the MetS group (p < 0.01 for both parameters). On the other hand, the MetS/E + EX shows an insignificant reduction in collagen deposition, revealing a higher fibrosis score (p = 0.75) and collagen area quantification (p = 0.72).

### 3.2 E1231 improves liver function, oxidative stress markers, and survival in a metabolic syndrome rat model

**FIGURE 3:**
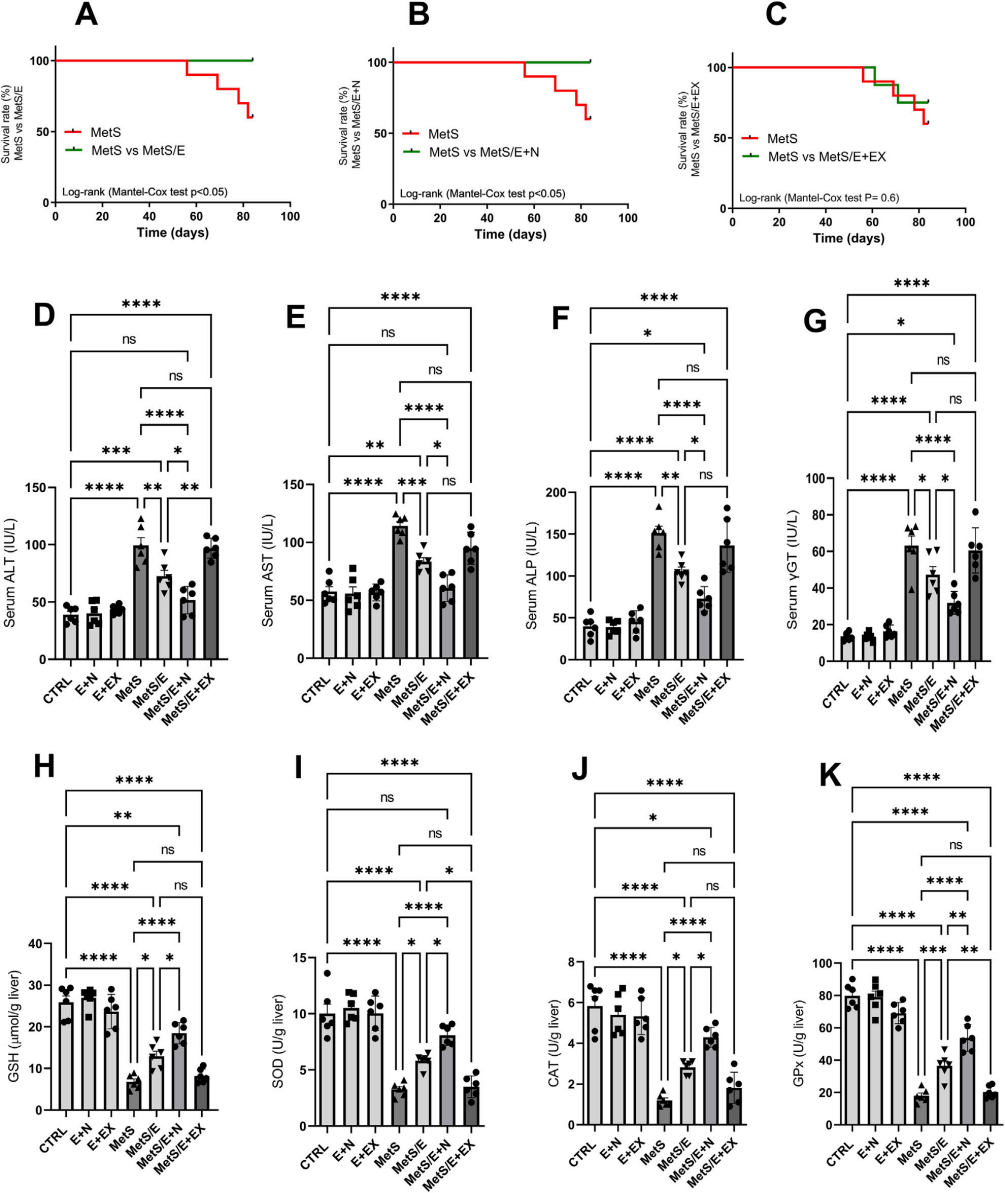
E1231 improves liver function, oxidative stress markers, and survival in a metabolic syndrome rat model. Survival analysis **(A–C)**, measurement of serum liver enzyme levels [ALT, AST, ALP, γGT; **(D–G)**], and oxidative stress markers [GSH, SOD, CAT, GPx; **(H–K)**]. Statistical analysis was performed using one-way ANOVA followed by Tukey’s *post hoc* test for biochemical markers and the Log-rank (Mantel-Cox) test for survival analysis, n = 6. *p < 0.05, **p < 0.01, ***p < 0.001, ****p < 0.0001.

As shown in Figure 3, the survival analysis (Panels A–C) shows a significant improvement in survival rates for both the MetS/E group (Panel A) compared to the MetS group (Log-rank (Mantel-Cox) test p < 0.05) and the MetS/E + N group (Panel B) compared to the MetS group (Log-rank (Mantel-Cox) test p < 0.05). However, no significant difference in survival rates was observed between the MetS and MetS/E + EX groups (Panel C). This indicates that while E1231 alone or in combination with NMN improves survival, the addition of the SIRT1 inhibitor EX527 does not significantly alter survival outcomes compared to the MetS group (Log-rank (Mantel-Cox) test p = 0.6). Compared to the CTRL group, the levels of ALT (D), AST (E), ALP (F), and γGT (G) are significantly elevated in the MetS group (p < 0.0001 for all). Treatment with E1231 in the MetS/E group significantly reduced ALT, AST, ALP, and γGT levels compared to the MetS group (p < 0.01, 0.001, 0.01, and 0.05, respectively). The MetS/E + N group showed an even greater reduction in these liver enzyme levels compared to the MetS group and showed a significant decrease in ALT, AST, ALP, and γGT levels compared to the MetS/E group (p < 0.0001 for all). In contrast, the MetS/E + EX group, which includes the SIRT1 inhibitor EX527, showed insignificant reductions in ALT, AST, ALP, and γGT levels compared to the MetS group (p = 0.99, 0.06, 0.76, and 0.99, respectively). Similarly, oxidative stress markers such as GSH (H), SOD (I), CAT (J), and GPx (K) are significantly decreased in the MetS group compared to the CTRL group (p < 0.0001 for all). E1231 treatment in the MetS/E group significantly increases the levels of GSH, SOD, CAT, and GPx compared to the MetS group (p < 0.05, 0.05, 0.05, and 0.001, respectively). The MetS/E + N group further enhances these antioxidative markers with significant increases compared to both the MetS group (p < 0.0001 for all) and the MetS/E group (p < 0.05, 0.05, 0.05, and 0.01, respectively). However, in the MetS/E + EX group, no significant differences are observed when compared directly to the MetS group (p = 0.97, 0.99, 0.8, and 0.99, respectively).

### 3.3 E1231 improves lipid profiles and liver weight index in a metabolic syndrome rat model

**FIGURE 4:**
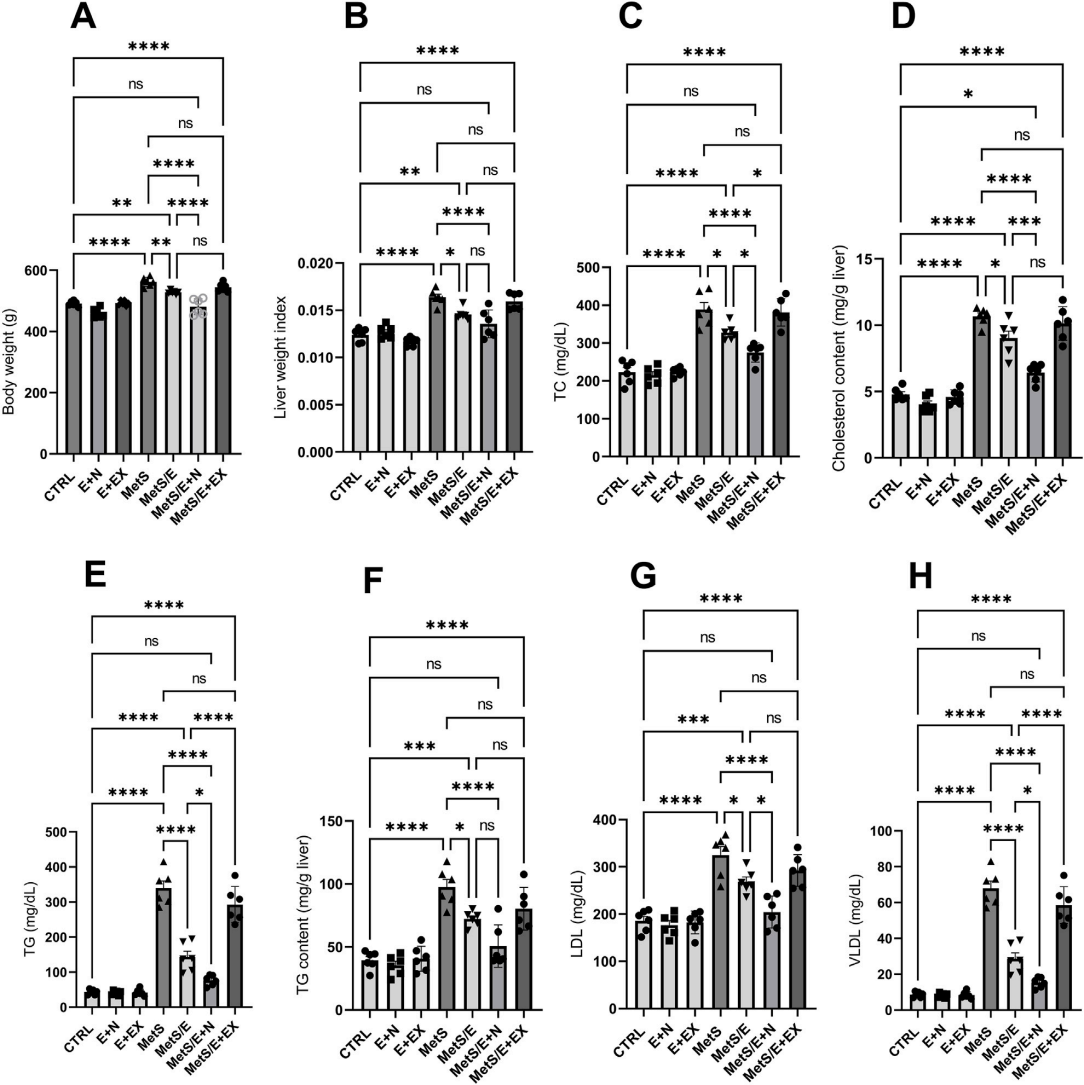
E1231 improves lipid profiles and liver weight index in a metabolic syndrome rat model. Analysis of body weight **(A)**, liver weight index **(B)**, and lipid profiles [total cholesterol, liver cholesterol content, triglycerides, liver triglyceride content, LDL, VLDL; **(C–H)**]. Statistical analysis was conducted using one-way ANOVA followed by Tukey’s *post hoc* test, n = 6. *p < 0.05, **p < 0.01, ***p < 0.001, ****p < 0.0001.

As depicted in Figure 4, compared to the CTRL group, the MetS group showed significant increases in body weight, liver weight index, TC, liver cholesterol content, TG, liver TG content, LDL, and VLDL (p < 0.0001 for all). Treatment with E1231 in the MetS/E group results in significant reductions in all these parameters compared to the MetS group (p < 0.01, 0.05, 0.05, 0.05, 0.0001, 0.05, 0.05 and 0.0001, respectively). The MetS/E + N group showed an even more significant reduction than the MetS group (p < 0.0001 for all). Also, it showed a significant decrease in body weight, TC, liver cholesterol content, TG, LDL, and VLDL compared to the MetS/E group (p < 0.0001, 0.05, 0.001, 0.05, 0.05, and 0.05, respectively). In contrast, the MetS/E + EX group, which includes the SIRT1 inhibitor EX527, showed an insignificant change in body weight, liver weight index, TC, liver cholesterol content, TG, liver TG content, LDL, and VLDL compared to the MetS group (p = 0.35, 0.97, 0.99, 0.92, 0.16, 0.21, 0.52, and 0.16 respectively).

### 3.4 E1231 counteracts the effect of HFD on body weight over weeks, showing anti-obesity effects

**FIGURE 5:**
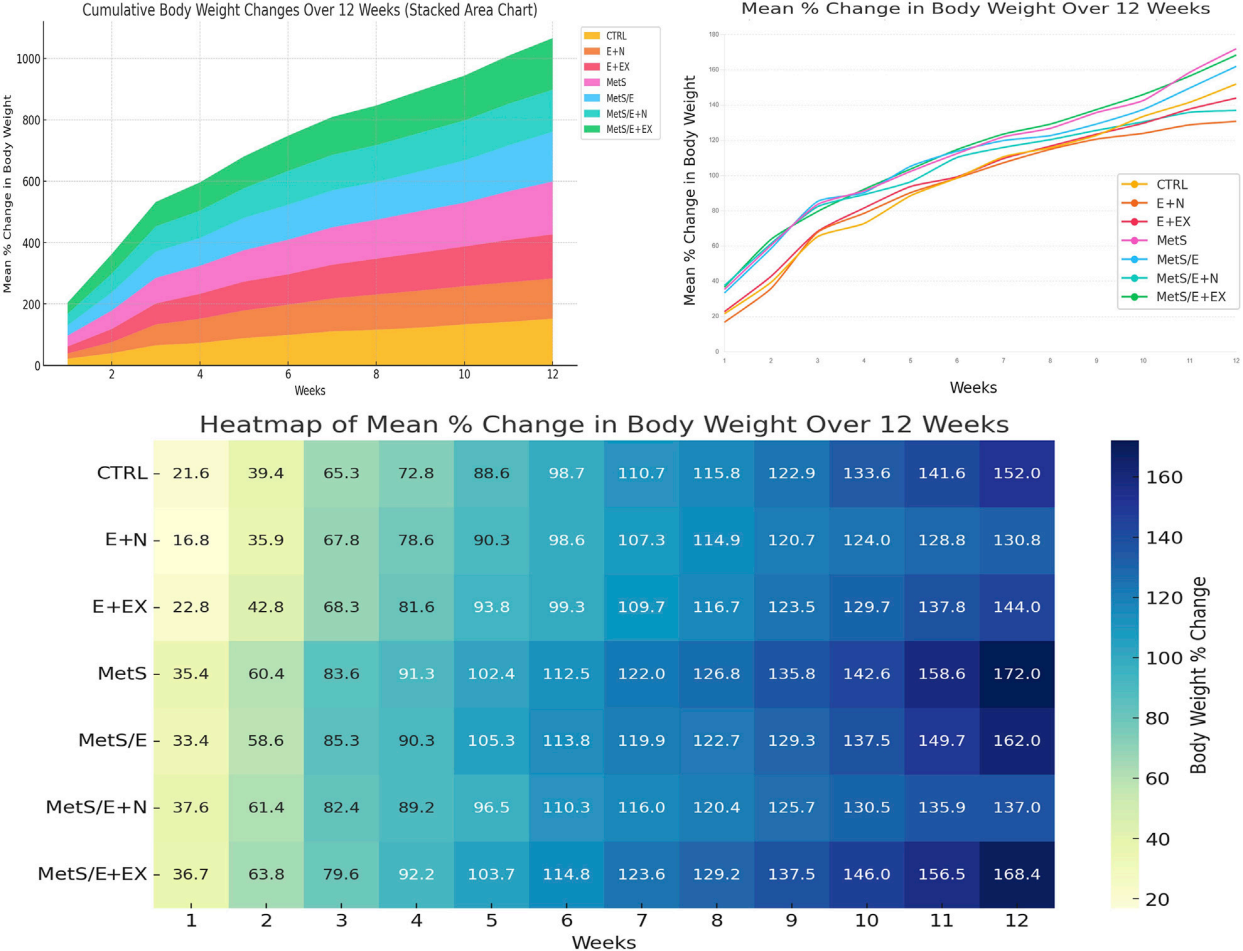
The stacked area chart upper left panel displays the cumulative weight changes over time. The line plot upper right panel tracks the mean percentage change in body weight over time to highlight the advancing weight gain in the MetS group and the partial mitigation achieved by treatments. The heatmap lower panel provides a detailed overview of the weekly changes in which darker shades indicate greater weight gain.

As displayed in Figure 5 (lower panel), the heatmap visualization displays trends in weight changes across different experimental groups over 12 weeks. Groups fed with an HFD (MetS and MetS/E + EX) demonstrated consistently higher percentages of weight gain compared to control groups fed on SD (CTRL, E + N, and E + EX) or treatment groups (MetS/E and MetS/E + N). Color intensities reflect the magnitude of weight change. Darker shades indicate undue increases. Notably, MetS and related groups exhibited the highest weight gain. This finding confirms the impact of the HFD. Groups receiving interventions (MetS/E and MetS/E + N) demonstrated gradual but reduced weight gains over time, with the MetS/E + N demonstrating the most significant anti-obesity effects. The line plot (upper right panel) depicts the dynamic progression of body weight changes in each group over weeks. The MetS and MetS/E + EX groups underwent the steepest increase in weight. In contrast, the control groups (CTRL, E + N, and E + EX) showed a more gradual weight gain over time. The intervention groups (MetS/E + MetS/E + N) exhibit moderate increases. This hints at partial mitigation of weight gain. This visualization highlights the effectiveness of the interventions in counteracting obesity. The stacked area chart (upper left panel) provides a cumulative view of the body weight changes over weeks. This type of chart confirms the effectiveness of HFD in promoting obesity. The MetS and MetS/E + EX groups influenced the largest area of the cumulative plot. This mirrors the highest weight gain throughout the experimental period. Groups receiving combined interventions (e.g., MetS/E and, in particular, MetS/E + N) displayed little contribution to the cumulative weight increase. This result reveals that these treatments partially countered the effects of HFD. Control groups (CTRL, E + N, and E + EX) maintained a relatively low trajectory. This is consistent with the expected weight gain on a standard diet. These cumulative trends highlight the asserted influence of the HFD and the beneficial effect of interventions (E1231 and E1231+NMN) in mitigating weight gain over time.

### 3.5 E1231 improves blood pressure, glucose homeostasis, and renal function in a metabolic syndrome rat model

**FIGURE 6:**
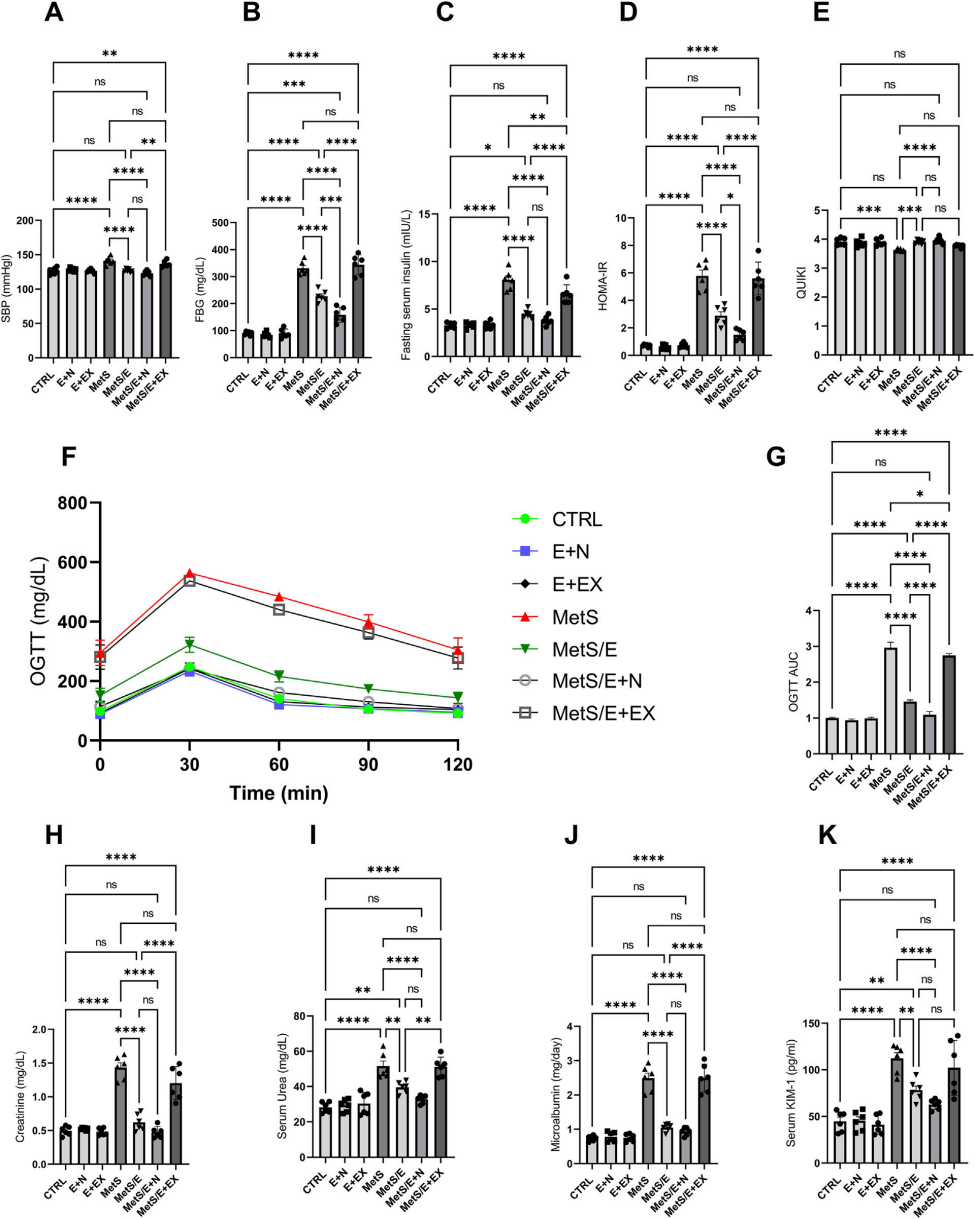
E1231 improves blood pressure, glucose homeostasis, and renal function in a metabolic syndrome rat model. Measurements of systolic blood pressure (SBP; **(A)**, fasting blood glucose (FBG; **(B)**, fasting serum insulin **(C)**, HOMA-IR **(D)**, OGTT AUC **(E)**, and renal function markers (creatinine, urea, microalbumin, KIM-1; **(F–K)** Statistical analysis was performed using one-way ANOVA followed by Tukey’s *post hoc* test, n = 6. *p < 0.05, **p < 0.01, ***p < 0.001, ****p < 0.0001.

As displayed in Figure 6, compared to the CTRL group, the MetS group showed significant increases in SBP, FBG, fasting serum insulin, HOMA-IR, OGTT AUC, serum creatinine, serum urea, microalbumin, and serum KIM-1 (p < 0.0001 for all). In contrast, it showed a significant decrease in QUIKI (p < 0.001). Treatment with E1231 in the MetS/E group results in significant reductions in SBP, FBG, fasting serum insulin, HOMA-IR, OGTT AUC, serum creatinine, and microalbumin (p < 0.0001 for all). For serum urea and serum KIM-1, the MetS/E group demonstrated significant reductions and showed p < 0.01 compared to the MetS group. On the other hand, the MetS/E group demonstrated a significant increase in QUIKI and showed p < 0.001 compared to the MetS group.

The MetS/E + N group showed an even more significant decrease in the parameters mentioned, except QUIKI, compared to the MetS group (p < 0.0001 for all). Also, it showed a significant reduction in FBG, HOMA-IR, and OGTT AUC compared to the MetS/E group (p < 0.001, 0.05, and 0.0001, respectively). In contrast, the MetS/E + EX group, which includes the SIRT1 inhibitor EX527, showed insignificant change in these parameters compared to the MetS group, except for fasting serum insulin and OGTT AUC (p < 0.01 and 0.05). Regarding the QUIKI, The MetS/E + N group showed a significant increase compared to the MetS group (p < 0.0001). In contrast, the MetS/E + EX group showed an insignificant change in QUIKI compared to the MetS group (p = 0.2).

### 3.6 E1231 modulates inflammatory, fibrotic, and insulin signaling pathways in a metabolic syndrome rat model

**FIGURE 7:**
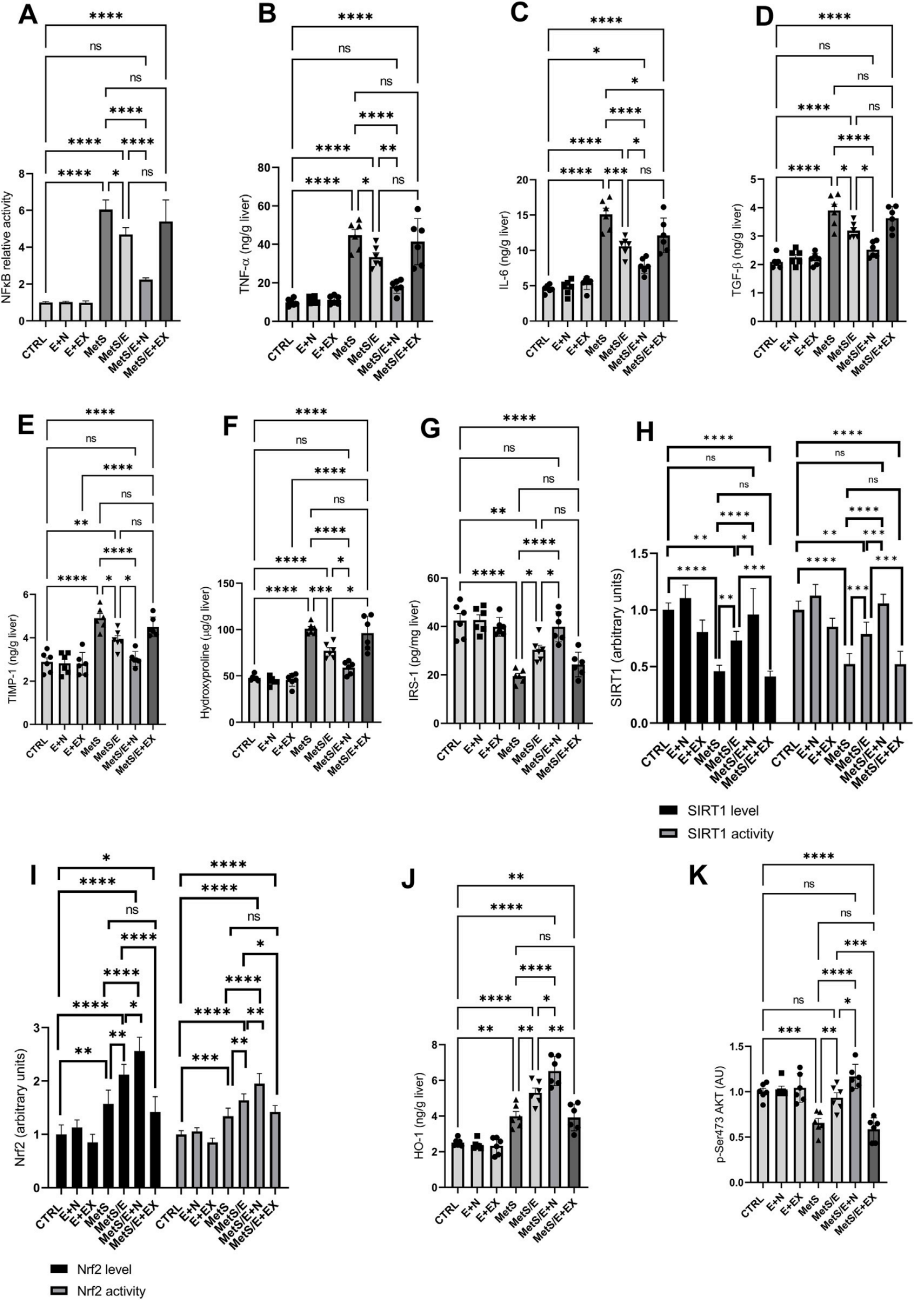
E1231 modulates inflammatory, fibrotic, and insulin signaling pathways in a metabolic syndrome rat model. Analysis of inflammatory markers [NF-κB, TNF-α, IL-6, TGF-β1; **(A–D)**], fibrosis indicators [TIMP-1, hydroxyproline; **(E, F)**], insulin signaling pathways [IRS-1, p-Ser473 AKT; **(G, K)**], and protective signaling molecules [SIRT1 activity, Nrf2 activity, HO-1; **(H–J)**]. Statistical analysis was conducted using one-way ANOVA followed by Tukey’s *post hoc* test, n = 6. *p < 0.05, **p < 0.01, ***p < 0.001, ****p < 0.0001.

As depicted in Figure 7, compared to the CTRL group, the MetS group showed significant increases in NFκB activity (p < 0.0001), TNF-α (p < 0.0001), IL-6 (p < 0.0001), TGF-β1 (p < 0.0001), TIMP-1 (p < 0.0001), hydroxyproline (p < 0.0001), Nrf2 level (p < 0.01) and activity (p < 0.001), and HO-1 (p < 0.01) along with decreased SIRT1 level (p < 0.0001) and activity (p < 0.0001), IRS-1 (p < 0.0001), and p-Ser473 AKT (p < 0.001). Treatment with E1231 in the MetS/E group results in significant reductions in NFκB activity (p < 0.05), TNF-α (p < 0.05), IL-6 (p < 0.001), TGF-β1 (p < 0.05), TIMP-1 (p < 0.05), and hydroxyproline levels (p < 0.001) compared to the MetS group. Additionally, IRS-1 (p < 0.05), p-Ser473 AKT (p < 0.01), SIRT1 level (p < 0.01) and activity (p < 0.001), Nrf2 level (p < 0.01) and activity (p < 0.01), and HO-1 (p < 0.01) are significantly increased in the MetS/E group compared to the MetS group. The MetS/E + N group showed further improvements in these parameters compared to the MetS group (p < 0.0001 for all) and MetS/E group (NFκB activity, p < 0.0001; TNF-α, p < 0.01; IL-6, p < 0.05); TGF-β1, p < 0.05; TIMP-1, p < 0.05; hydroxyproline, p < 0.05; Nrf2 level, p < 0.05 and activity, p < 0.01; HO-1, p < 0.05; IRS-1, p < 0.05; p-Ser473 AKT, p < 0.05; SIRT1 level, p < 0.05 and activity, p < 0.001. However, these beneficial effects are attenuated in the MetS/E + EX group (non-significant values except for IL-6, which showed p < 0.05).

### 3.7 Correlation analysis of key metabolic and inflammatory markers in a metabolic syndrome rat model treated with E1231

**FIGURE 8:**
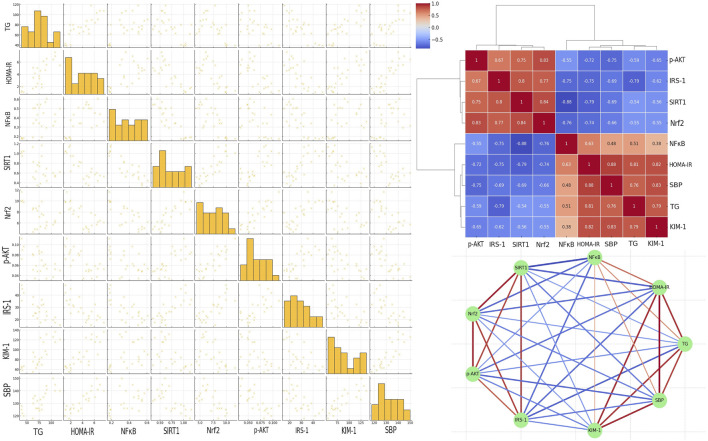
Correlation analysis of key markers in a metabolic syndrome rat model treated with E1231. The scatter plot matrix is shown in the left panel. The clustered heatmap is displayed in the upper right panel. The network analysis of Pearson correlation coefficients is illustrated in the lower right panel.

Figure 8 (upper right panel) presents a detailed correlation analysis among key metabolic, inflammatory, and signaling markers, including p-AKT, across the different experimental groups treated with E1231. The heatmap of Pearson correlation coefficients reveals strong positive correlations between HOMA-IR and SBP (r = 0.88), KIM-1 and SBP (r = 0.83), and HOMA-IR and TG (r = 0.81). Additionally, p-AKT shows positive correlations with SIRT1 (r = 0.75), Nrf2 (r = 0.83), and IRS-1 (r = 0.67), indicating that the activation of p-AKT is associated with higher levels of SIRT1 and Nrf2, which are protective against metabolic dysfunction. Strong negative correlations are observed between SIRT1 and NFκB (r = −0.88), SIRT1 and HOMA-IR (r = −0.79), SIRT1 and SBP (r = −0.69), as well as between Nrf2 and NFκB (r = −0.76), Nrf2 and HOMA-IR (r = −0.74), and Nrf2 and SBP (r = −0.66). p-AKT also shows negative correlations with TG (r = −0.59), NFκB (r = −0.55), HOMA-IR (r = −0.72), and SBP (r = −0.75), suggesting that the activation of p-AKT, along with SIRT1 and Nrf2, is inversely related to markers of inflammation and metabolic dysfunction. The network analysis (lower right panel) further highlights these relationships, with strong negative correlations (blue lines) between SIRT1, Nrf2, and p-AKT with markers like NFκB, HOMA-IR, and SBP, reinforcing the protective role of these signaling pathways in the context of metabolic syndrome. Conversely, strong positive correlations (red lines) between HOMA-IR, SBP, TG, and KIM-1 emphasize their interconnectedness in driving the pathological changes observed in metabolic syndrome. These findings strongly support the protective effects of E1231, with the activation of p-AKT, along with increased SIRT1 and Nrf2 activity, playing a central role in mitigating inflammation, insulin resistance, and associated metabolic disturbances. The left panel represents a scatter plot matrix demonstrating the pairwise relationships between parameters analyzed, confirming correlations in the heatmap. The diagonal histograms show parameter distributions. The off-diagonal scatter plots reveal trends. The clustering of points reflects strong correlations, while more dispersed plots indicate weaker correlations. This visual summary reinforces the protective effects of SIRT1, Nrf2, and p-AKT.

### 3.8 Impact of key metabolic and molecular parameters on metabolic syndrome measured as HOMA-IR

**FIGURE 9:**
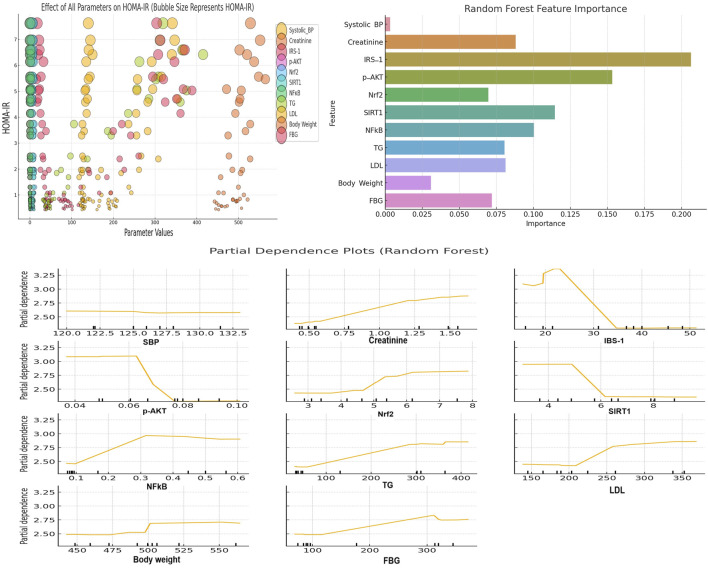
The upper left panel depicts a bubble plot showing the impact of different parameters on HOMA-IR (a measure of metabolic syndrome). The x-axis shows the parameter values and the y-axis shows HOMA-IR levels. Bubble sizes correspond to the magnitude of HOMA-IR. Larger bubbles indicate higher HOMA-IR values. The upper right panel shows the Random Forest feature importance plot. This plot highlights the parameters that most significantly contribute to predicting HOMA-IR (metabolic syndrome severity). The lower panel presents PDPs for key parameters from the Random Forest model. This plot illustrates the specific influence of various parameters on metabolic syndrome. Specific threshold values for each parameter are visualized in the PDPs.

The bubble plot in Figure 9 (upper left panel) illustrates the impact of multiple parameters on HOMA-IR, which is commonly used as a determinant of metabolic syndrome. Elevated creatinine is associated with increased HOMA-IR. Increased NFκB also correlates with higher HOMA-IR. Additionally, the insulin signaling molecules IRS-1 and p-AKT display inverse relationships with HOMA-IR. Similarly, SIRT1 shows protective effects by reducing HOMA-IR. Furthermore, higher TG, LDL, body weight and FBG levels consistently align with larger HOMA-IR bubbles. In contrast, higher SBP shows inconsistent relationships with HOMA-IR. Furthermore, higher Nrf2 values do not consistently correspond to lower HOMA-IR values.

### 3.9 Random forest machine learning algorithm revealed the importance of IRS-1, p-AKT, SIRT1, and NFκB for predicting metabolic syndrome severity

The Random Forest feature importance plot (Figure 9; upper right panel) provided insights into which features (parameters) are the most important for predicting HOMA-IR (metabolic syndrome severity). The feature importance plot revealed that parameters such as IRS-1, p-AKT, SIRT1, and NFκB are the most significant contributors to the prediction of HOMA-IR. This finding highlights their significance in determining metabolic syndrome severity.

### 3.10 Partial dependence plot analysis of key parameters affecting metabolic syndrome severity

The PDPs depicted in Figure 9 (lower panel) provide insights into how various parameters influence metabolic syndrome in a Random Forest model. Creatinine displays a slightly positive trend with HOMA-IR, with a noticeable rise after the threshold of 1.0 mg/dL. IRS-1, SIRT1, and p-AKT demonstrate inverse solid relationships, with significant drops in HOMA-IR when IRS-1 levels exceed 30, SIRT1 exceeds 6, and p-AKT reaches around 0.07. This suggests that these parameters contribute to alleviating the severity of metabolic syndrome at these thresholds. NFκB shows a positive relationship with HOMA-IR, with a threshold effect of around 0.3. This finding indicates the role of NFκB in worsening metabolic syndrome at higher levels. Both TG and LDL display clear positive associations with HOMA-IR, with noticeable effects when TG exceeds 150 mg/dL and LDL exceeds 250 mg/dL. Additionally, body weight and FBG show positive relationships. In this context, a threshold of influence was observed around 500 g for body weight (obesity) and 150 mg/dL for FBG, reinforcing their contributions to increased metabolic syndrome severity. Furthermore, SBP shows little impact on HOMA-IR by showing a flat relationship across the range of values. Lastly, Nrf2 demonstrates a positive trend, where higher levels are associated with increased HOMA-IR. It seems that this Nrf2 result is contrary to expectations. However, Nrf2 elevation might be attributed to a compensatory response to heightened oxidative stress associated with metabolic syndrome.

## 4 Discussion

Metabolic syndrome is a complex disorder that increases the risk of developing type 2 diabetes and other related complications (Alemany, 2024). Current therapies frequently fail to address the underlying molecular pathways leading to the condition. Indeed, physicians often take advantage of using multiple drugs to manage every individual component of the syndrome (Lillich et al., 2021). This polypharmacy exposes patients with metabolic diseases to adverse effects and drug-drug interactions. Therefore, in this study, we aimed to investigate the potential of the SIRT1 activator E1231 as a new single therapy for managing metabolic syndrome. Additionally, we evaluated the potential of NMN when combined with E1231. Given that NMN is a precursor to NAD^+^, we hypothesized that NMN would presumably enhance the efficacy of E1231 by ensuring a supply of this crucial coenzyme.

Our histological examination of the MetsS rat group revealed liver injury that is consistent with NAFLD, which is among the complications of metabolic syndrome. When combined with NMN, E1231 resulted in the most significant improvement. NMN supplementation likely enhanced the effectiveness of SIRT1 activation, as indicated by decreased lobular inflammation and steatosis. It is important to note that our model only produced a mild form of fibrosis, and the liver tissues did not exhibit NASH features. However, SIRT1 activation significantly reduced fibrosis markers, as evidenced by decreased collagen deposition and hydroxyproline content. These effects might be ascribed to the downregulation of the pro-fibrotic mediators such as TGF-β1 and TIMP-1. Furthermore, the E1231 antifibrotic role is also supported by enhancing antioxidant responses, which mitigate the oxidative stress that drives fibrogenesis.

A significant prolongation of the survival rate accompanied the histological improvements. Survival analysis indicated that MetS significantly reduced the lifespan of the rats. Metabolic disturbances and chronic inflammation might have a role in the observed mortality. E1231 therapy improved survival rates. This improvement in survival correlates with the enhanced liver function and antioxidant defense observed in rats treated with E1231 or its combination with NMN. In this context, SIRT1 activation led to increased levels of key antioxidant enzymes (HO-1, GSH, SOD, CAT, and GPx). The activation of Nrf2 likely mediates this enhancement as it has been acknowledged that Nrf2 plays a central role in the cellular antioxidant response (Powers et al., 2024).

The liver weight index is a marker of hepatomegaly and steatosis. Consistent with the observed liver damage, the liver weight index was significantly elevated in the MetS group. E1231 alone, particularly in the MetS/E + N groups, significantly reduced the liver weight index and improved lipid profile. E1231 effectively reduced total cholesterol, triglycerides, LDL, and VLDL levels. Additionally, it decreased hepatic lipid accumulation. Furthermore, our model revealed significant disturbances in glucose homeostasis as indicated by elevated fasting blood glucose, fasting serum insulin, HOMA-IR, and OGTT AUC in the MetS group. SIRT1 activation by E1231 significantly improved these parameters. The improvement in glucose homeostasis is consistent with the observed upregulation of AKT. It has been recognized that AKT is critical for insulin signaling and glucose uptake (Savova et al., 2023). The lack of significant improvement when E1231 was combined with EX527 further underscores the importance of SIRT1 in regulating glucose metabolism.

In our study, elevated serum creatinine, urea, microalbumin, and KIM-1 indicate kidney impairment, a common metabolic syndrome complication. SIRT1 activation by E1231 significantly improved kidney function. This improvement is likely to be due to the reduction in oxidative stress and inflammation. In this regard, our results revealed decreased NFκB activity and enhanced Nrf2 signaling.

In this study, correlation analyses provided further insights into the protective role of SIRT1 activation in metabolic syndrome. SIRT1 was found to negatively correlate with NFκB, insulin resistance, and oxidative stress. This reveals that SIRT1 counteracts the harmful effects of these pathways. In addition, positive correlations were unveiled between SIRT1, p-AKT, and Nrf2 activity. This suggests that these signals can promote metabolic health by promoting insulin signaling to reduce oxidative stress and suppress inflammation. The strong interconnectedness between these molecules underscores the centrality of SIRT1 in coordinating a multifaceted protective response counteracting metabolic syndrome.

It was discovered that augmenting SIRT1 activity promotes phosphorylation and activation of AKT kinase through its deacetylation (Pillai et al., 2014). AKT, in turn, functions as a key regulator of glucose and lipid metabolism. Heightened AKT activity improves insulin sensitivity and protects against obesity and diabetes associated with metabolic syndrome. Conspicuously, IRS-1 is another critical component of the insulin signaling pathway. It triggers the signaling cascade, resulting in Akt activation (Martínez Báez et al., 2024). Additionally, Nrf2 is a transcription factor that modifies the antioxidant response (Abdelhamid et al., 2022; Cavalu et al., 2022). In this context, SIRT1 activates Nrf2 through deacetylation (Ding et al., 2016; Ren et al., 2019). This deacetylation process allows Nrf2 nuclear translocation to subsequently induce the expression of antioxidant and detoxification genes (Ren et al., 2019). This SIRT1-induced Nrf2 activation helps mitigate oxidative stress associated with metabolic syndrome. In addition, NFκB is a well-known pro-inflammatory transcription factor (Nasr et al., 2022). Again, SIRT1 suppresses NFκB signaling through the deacetylation of the p65 subunit (Hah et al., 2014; Ren et al., 2019). This SIRT1-induced NFκB suppression prevents the induction and the expression of inflammatory cytokines that promote insulin resistance and other complications of metabolic syndrome. The regulation of downstream gene transcription includes those encoding TNF-α and IL-6 (Guo et al., 2014; Hah et al., 2014).

It has been discovered that NMN supplementation activates SIRT1 on its own and mimics some of the effects of direct SIRT1 activators (Gomes et al., 2013; Wang et al., 2021). When combined, E1231, a direct SIRT1 activator, and NMN, an indirect SIRT1 activator might produce a more robust activation of SIRT1 through NAD^+^ augmentation. Therefore, both molecules may protect tissues on multiple fronts. They can provide stronger, more comprehensive metabolic benefits than single therapies. Furthermore, to reduce the risk of adverse events, lower effective doses of each compound may potentially be used.

In this study, Random Forest and PDPs highlight the critical role of certain metabolic and molecular parameters in influencing metabolic syndrome. It should be noted that HOMA-IR is commonly used as a determinant for metabolic syndrome (de Abreu et al., 2017; Dundar et al., 2023; Yuan et al., 2024). The Random Forest machine learning algorithm revealed that IRS-1, p-AKT, SIRT1, and NFκB are critical predictors of metabolic syndrome severity. Furthermore, PDPs provided detailed insights into how the individual parameters IRS-1, SIRT1, and p-AKT exhibit strong inverse relationships with metabolic syndrome. This suggests the protective roles of these parameters, especially beyond certain thresholds as revealed by the plots. Conversely, NFκB, TG, LDL, and FBG demonstrated positive associations with HOMA-IR. In this regard, clear threshold effects underlined their contributions to worsening metabolic syndrome. Interestingly, Nrf2 displayed a positive trend with HOMA-IR, which may suggest compensatory upregulation in response to oxidative stress rather than a direct protective effect. It has been acknowledged that Nrf2 plays an essential role in a cell’s ability to adapt to stress. Pathophysiological conditions quickly augment its expression in all organs (Maines et al., 1999). Furthermore, Nrf2 responds extremely to all types of stimuli that result in oxidative stress (Maines, 1988; Cavalu et al., 2022). These findings explain the increased Nrf2 and HO-1 levels found in metabolic syndrome in our study.

In addition to these insights, our results demonstrate progressive weight gain in HFD-fed groups, confirming its role in inducing obesity and exacerbating metabolic syndrome. In contrast, interventions with E1231 or E1231+NMN mitigated weight gain and demonstrated anti-obesity effects. These cumulative findings reinforce the relationship between obesity, insulin resistance, and metabolic syndrome severity.

## 5 Conclusion

Our findings revealed that E1231-induced SIRT1 activation protects against metabolic syndrome by modulating multiple signals. It enhanced IRS-1 upstream of AKT and activated AKT. SIRT1 also activated the antioxidant transcription factor Nrf2, inducing the expression of cytoprotective genes to mitigate oxidative stress associated with metabolic syndrome. Furthermore, SIRT1 suppressed the pro-inflammatory NFκB pathway. Thereby, E1231 prevented NFκB-induced expression of inflammatory cytokines that promote metabolic syndrome complications. Therefore, we can hypothesize that SIRT1 protects against metabolic syndrome through the coordinated effects on AKT, Nrf2, and NFκB signaling. Moreover, findings of this study suggest that E1231-driven SIRT1 activation represents a novel strategy for managing metabolic syndrome. Furthermore, exploring the effects of SIRT1 activators and other agents, such as NMN, may enhance treatment efficacy.

This combination therapy provides a new avenue for tackling the complexities of metabolic syndrome. Despite the valuable results, the rat model used in this study may not fully replicate the complexity of human metabolic syndrome. Hence, translating these findings into clinical settings should require further investigations. Besides, the long-term effects of SIRT1 activation were not assessed. Prolonged SIRT1 activation might result in unforeseen adverse events. Therefore, future studies should explore the long-term safety and efficacy of E1231 and NMN.

## Data Availability

The original contributions presented in the study are included in the article/supplementary material, further inquiries can be directed to the corresponding authors.
